# The effect of attention on body size adaptation and body dissatisfaction

**DOI:** 10.1098/rsos.211718

**Published:** 2022-02-23

**Authors:** T. House, I. D. Stephen, I. S. Penton-Voak, K. R. Brooks

**Affiliations:** ^1^ School of Psychological Science, Faculty of Medicine, Health and Human Sciences, Macquarie University, Sydney, Australia; ^2^ Department of Psychology, University of Bristol, Bristol, UK; ^3^ Department of Psychology, Nottingham Trent University, Nottingham, UK; ^4^ National Institute for Health Research Bristol Biomedical Research Centre, University Hospitals Bristol NHS Foundation Trust and University of Bristol, UK

**Keywords:** attention, attention training, adaptation, body dissatisfaction, body size, dot probe

## Abstract

Attentional bias to low-fat bodies is thought to be associated with body dissatisfaction—a symptom and risk factor of eating disorders. However, the causal nature of this relationship is unclear. In three preregistered experiments, we trained 370 women to attend towards either high- or low-fat body stimuli using an attention training dot probe task. For each experiment, we analysed the effect of the attention training on (i) attention to subsequently presented high- versus low-fat body stimuli, (ii) visual adaptation to body size, and (iii) body dissatisfaction. The attention training had no effect on attention towards high- or low-fat bodies in an online setting (Experiment 1), but did increase attention to high-fat bodies in a laboratory setting (Experiment 2). Neither perceptions of a ‘normal’ body size nor levels of body dissatisfaction changed as a result of the attention training in either setting. The results in the online setting did not change when we reduced the stimulus onset-asynchrony of the dot probe task from 500 to 100 ms (Experiment 3). Our results provide no evidence that the dot probe training task used here has robust effects on attention to body size, body image disturbance or body dissatisfaction.

## 1. Introduction

Body image disturbance is a multifaceted construct associated with negative health consequences. The perceptual component of body image disturbance is referred to as body size and shape misperception and is presented when a person over- or under-estimates their body size [1]. The attitudinal component of body image disturbance is referred to as body dissatisfaction and is defined as the negative subjective evaluation of one's body [2,3]. Given society's widespread adoption of the thin-ideal [4–6], the two constructs are probably related, because the overestimation of one's own body size might cause feelings of body dissatisfaction. Further, both constructs are associated with eating disorders. For example, the overestimation of one's body size is a diagnostic symptom of anorexia nervosa [7], as well as a core feature of bulimia nervosa [8,9]. Body dissatisfaction is a risk factor for eating disorders such as anorexia nervosa and bulimia nervosa [3], and possibly for binge eating disorder and purging disorder [10]. Body dissatisfaction is also a diagnostic symptom of anorexia nervosa [7]. Therefore, both body dissatisfaction and body size and shape misperception are important constructs to consider in the design of eating disorder interventions.

A potential mechanism involved in the development and maintenance of body size and shape misperception is a visual adaptation, which is the temporary perceptual shift experienced after exposure to extreme stimuli [1,11]. When applied to body size perception, exposure to low (high)-fat body stimuli causes people to overestimate (underestimate) the body fat of subsequently presented body stimuli. These perceptual shifts are called body size after-effects and have been repeatedly demonstrated in experiments measuring the change in the body size that participants perceive to be ‘normal’ [12–14]. In these experiments, participants who adapt to low (high)-fat bodies perceive subsequently seen bodies to be larger (smaller) than they really are, including stimuli that they would previously have seen as normal. As such, they need to reduce (increase) the size of bodies when selecting normal-looking stimuli post-adaptation. Importantly, the current perception of the body stimuli becomes distorted by adaptation, and not the representation of the body stored in memory [15,16]. A possible negative consequence of body size adaptation is the misperception of one's own body size. Brooks *et al*. [12] found that participants exposed to contracted (expanded) unfamiliar bodies for 2 min subsequently overestimated (underestimated) their own body size. Further, Salvato *et al*. [17] found that participants exposed to images of high-fat unfamiliar bodies proceeded to strongly associate ‘thin’ and ‘self’ concepts on the Implicit Association Test, unlike participants exposed towards images of low-fat unfamiliar bodies who demonstrated a significantly weaker association between ‘thin’ and 'self’ concepts.

Body size adaptation is also indirectly related to body dissatisfaction, with this relationship being mediated by visual attention. Eye-tracking [18–21] and reaction time [22–24] studies show that people with high body dissatisfaction direct more attention towards low-fat body stimuli than people with low body dissatisfaction. Further, Stephen and colleagues demonstrated that people adapt to the body size they direct more attention towards. When presented with pairs of bodies, one low and one high in body fat, people with higher body dissatisfaction directed more attention towards low-fat bodies, and this attentional bias resulted in an overestimation of the size of subsequently presented body stimuli [25].

Cognitive behavioural theories suggest that an attentional bias towards low-fat bodies is both a cause and a consequence of body dissatisfaction [26]. A possible causal pathway could be that directing more attention towards low-fat bodies leads a person to overestimate their body size due to body size adaptation, and this overestimation increases feelings of body dissatisfaction. This suggestion is supported by Bould and colleagues [27] who found that women exposed to unfamiliar high-fat body stimuli reported reduced size estimations for their own body, as well as reduced body dissatisfaction. While these observations demonstrate that body size adaptation can influence a person's body dissatisfaction, such effects do not always materialize. For example, Stephen and colleagues presented participants simultaneously with high- and low-fat body stimuli and instructed separate groups to fixate their eyes on one body type or the other. Although participants adapted to the body size to which they were instructed to attend, participants' body dissatisfaction did not change [28]. One possible explanation for the discrepancy in findings is that while Stephen and colleagues effectively manipulated overt attention, the participants may have fixated on their designated body size while simultaneously covertly attending to the contrasting body size in their peripheral vision [28,29]. This may also explain why the body size after-effects found by Stephen and colleagues (*d* = 0.42 and *d* = 0.63) were smaller than those found in similar adaptation studies that presented participants with only one body type (e.g. *d* = 1.86 and *d* = 2.15; [30]).

An alternative method of attention modification is the training dot probe task, which involves presenting participants with a pair of stimuli followed by a probe that the participants respond to as quickly as possible [31]. While the pair of stimuli is visible, the participant is free to attend (overtly or covertly) to either stimulus. During training, the probe replaces one stimulus type on 100% of the trials, which increases attention to this stimulus type. This change in attention is measured using participants’ reaction times on a pre- and post-training assessment version of the dot probe task, in which the probe has an equal probability of replacing each stimulus type. The training dot probe task has received considerable attention because, if therapeutic effects can be demonstrated, the task is low in cost and intensity and can potentially be administered online without a therapist present [32]. Meta-analyses show that the training dot probe task can be used to train participants to attend away from threatening stimuli (e.g. angry faces), resulting in reduced symptoms of anxiety [33,34]. However, the effect sizes for symptom reduction are likely to be small [35].

Dondzilo and colleagues used a training dot probe task in which the probe replaced a low-fat body stimulus (rather than neutral abstract art stimulus) on 100% of the training trials. As a result of the training, participants increased their attention towards low-fat bodies, as demonstrated by faster reaction times for probes replacing low-fat bodies at post-training than pre-training [36]. Although the diversion of covert attention away from the low-fat bodies may have been a possibility in Stephen and colleagues' study [28], this is unlikely to have been the case for participants in Dondzilo and colleagues’ study. Eye-movements are possible during the stimulus presentation in the dot probe task; however, the task is thought to be primarily a measure of covert attention [37]. The improved response speed displayed by participants suggests enhanced processing, which would have been unlikely if participants had been predominantly attending away from the low-fat body stimulus. The dot probe task may therefore be a more effective method of attention manipulation than simple instructions not to look at a given stimulus type.

Here, we aimed to test the causal effect of attention on body size adaptation and body dissatisfaction using a training dot probe task. For Experiment 1, we used an online dot probe task to train participants to attend towards either high- or low-fat body stimuli. We measured participants' attentional bias, body size adaptation and body dissatisfaction before and after the attention training. We hypothesized that participants trained to attend to low (high)-fat body stimuli would (1) increase their attention towards low (high)-fat body stimuli, (2) perceive lower (higher) fat subsequently presented body stimuli as ‘normal’, due to visual adaptation, and (3) increase (decrease) their body dissatisfaction.

## 2. Experiment 1

This experiment was preregistered on the Open Science Framework (doi:10.17605/OSF.IO/TJPZB).

### 2.1. Participants

We conducted a power analysis (G*Power v. 3.1.9.2 [38]) using the effect size reported by Dondzilo and colleagues (*d* = 0.49 [36]) which we reduced by a third to account for the inflation of published effect sizes (to *d* = 0.33). Based on the results, we recruited 150 participants (75 per condition) to provide the main analyses (one-sample *t*-tests) with 80% power to detect an effect for the primary outcome (change in attentional bias (ΔAB)) at an alpha level of 5%. We recruited White/European origin women aged 18–35 years (*M*_age_ = 23.95, s.d. = 5.22; *M*_BMI_ = 25.71, s.d. = 9.62). We placed no restrictions on the participant's country of residence. Sixty-six participants were recruited and reimbursed with £7.50 (GBP) via Prolific (www.prolific.co) and 84 participants were recruited and reimbursed with course credit via Macquarie University's study sign-up system. Participants were pseudorandomly allocated to each training condition to maintain even sample sizes across conditions.

### 2.2. Stimuli

**Figure 1.**
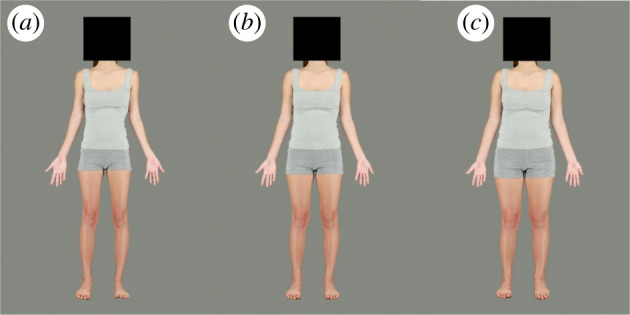
Example body stimuli; (*a*) shows the version of the target identity with lowest fat mass (Frame 0); (*b*) shows the unmanipulated version of the target identity (Frame 6); (*c*) shows the version of the target identity with the highest fat mass (Frame 12).

Twenty photographs of White/European origin women (*M*_age_ = 21.15, s.d. = 3.60; *M*_BMI_ = 20.15, s.d. = 1.23) were obtained from an existing database [39]. Each woman was photographed under standardized lighting conditions, with camera settings held constant, and wearing standard grey, tight-fitting singlets and shorts. Each target identity was transformed to produce a series of 13 frames, in which frame 0 was reduced by 12 kg of apparent body fat mass, increasing in steps of 2 kg of apparent fat mass per frame such that frame 6 was the original image, and frame 12 was increased by 12 kg of apparent fat mass [40]. These transforms have been used effectively to induce body size after-effects in previous studies [28,39]. The face of each target identity was obscured with a black square and the background was edited to a uniform grey (figure 1). The stimulus size depended on the participant's device screen size; however, the experiment was always presented in a display with a 4 : 3 aspect ratio and therefore the stimulus aspect ratios were the same for each participant. For the dot probe task, the body stimulus size was 30% of the display's width and 60% of the display's height. For the method of adjustment task, the body stimulus size was 35% of the display's width and 70% of the display's height.

### 2.3. Measures

#### 2.3.1. Dot probe task

**Figure 2.**
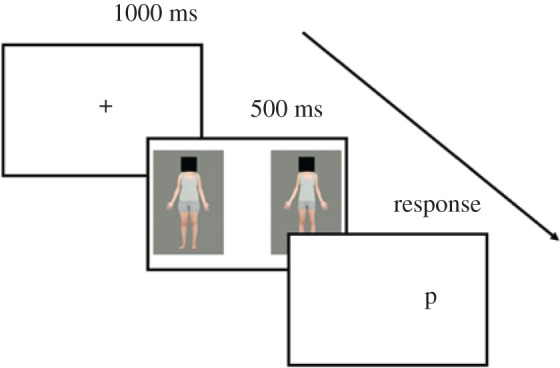
Example dot probe trial. Each dot probe trial started with a 1000 ms fixation, followed by one high- and one low-fat body stimulus presented for 500 ms. Then, a probe appeared (the letter ‘p’ or ‘q’) on either the left or right side of the screen. Participants had to identify the letter as quickly and accurately as possible. In this example trial, the probe (p) appeared in the same location as the low-fat body stimulus.

The dot probe task was adapted from Dondzilo and colleagues [22,36]. Following a 1000 ms fixation, two body stimuli were simultaneously presented for 500 ms. Body stimulus pairs consisted of the lowest and highest body fat frames (Frame 0 and Frame 12) of the same target identity with left/right position randomized. The centre of each body stimulus was located on the midpoint of the display's y-axis and 25% of the display's width away from the midpoint on the x-axis. Immediately after presentation of body stimuli, a random probe (either the letter ‘p’ or ‘q’) appeared in the position previously occupied by one of the pair. Participants were instructed to identify the letter as quickly and accurately as possible, by pressing the appropriate keys (p or q) on the keyboard. Once a response had been made, the next trial would begin immediately (figure 2).

For training dot probe trials, the location of the probe was dependent on the experimental condition. For participants trained to attend to high-fat body stimuli, the probe replaced the high-fat body stimulus on 100% of the training trials (vice versa for low-fat training). Participants completed 360 training dot probe trials, presented in 6 blocks of 60 trials with a 15 s break between each block. The training dot probe task used a set of 10 target identities presented in a randomized order for each participant.

To measure the change in attentional bias (ΔAB), participants completed 80 pre-training and 80 post-training dot probe trials. The probe location was randomized so that the probe had an equal probability of replacing each body stimulus. The body stimuli were a different set of 10 target identities to the training dot probe trials and were presented in a randomized order for each participant. To calculate the pre- and post-training attentional bias scores, we followed Dondzilo and colleagues' approach and excluded trials if the participant responded incorrectly, or if their reaction time was less than 200 ms or more than 2.5 standard deviations above the participant's mean reaction time on the pre- and post-training dot probe trials [22,36]. The mean reaction times of the remaining trials were substituted into the following formula [41]:

$$\mathrm{Attentional}\;\mathrm{bias}\;\mathrm{score}=\frac{\left([\mathrm{LPRT}\;\text{–}\;\mathrm{LPLT}]+[\mathrm{RPLT}\;\text{–}\;\mathrm{RPRT}]\right)}{2}.$$

For this formula, ‘L’ refers to the left side of the screen, ‘R’ refers to the right side of the screen, ‘P’ refers to the probe and ‘T’ refers to the target stimulus (for the purposes of our research the target stimulus was always the low-fat body). Therefore, the ‘LPRT’ refers to the mean response time when the probe (P) was located on the left (L) side but the low-fat body stimulus (T) was located on the right (R) side, and so on. A positive attentional bias score represents an attentional bias to low-fat body stimuli and a negative attentional bias score represents an attentional bias to high-fat body stimuli. ΔAB was calculated by subtracting the pre-training dot probe attentional bias score from the post-training dot probe attentional bias score. Therefore, a positive (negative) ΔAB meant that participants directed more attention toward low (high)-fat body stimuli after the training than before.

#### 2.3.2. Point of subjective normality

To measure body size adaptation, participants completed a modified version of the method of adjustment task [39]. In a given trial, participants were presented with one of the 13 frames, selected at random, for a single target identity, centred on the display. Participants could cycle through the 13 frames for the target identity by pressing ‘p’ on the computer keyboard to move to the next highest body fat frame and pressing ‘q’ on the keyboard to move to the next lowest body fat frame. The sequence was looped so participants were able to manipulate the target identity's body size by continually cycling through the 13 frames. Participants were presented with 10 target identities at both pre- and post-training. Participants were asked to manipulate the appearance of each body and select the image that they thought represented a normal-sized body. We did not specify the definition of a normal-sized body to participants, allowing them to use their own interpretation. The body stimuli were the same 10 target identities used in the pre- and post-training dot probe trials and therefore were a different set to those used in the training dot probe trials. Body stimuli were presented in a randomized order for each participant. The mean fat mass chosen as ‘normal-sized’ for the 10 target identities was used to calculate point of subjective normality (PSN) scores. Change in PSN (ΔPSN) was calculated by subtracting the pre-training PSN score from the post-training PSN score. A positive (negative) ΔPSN meant that the body size participants perceived to be ‘normal’ was higher (lower) after the training than before.

#### 2.3.3. Body dissatisfaction

Body dissatisfaction was measured using a modified version of the body shape satisfaction scale [42]. The scale required participants to rate their satisfaction with 18 parts or features of their body, including their waist, stomach and thighs. Participants were asked to respond based on their feelings ‘at this moment’ to specifically measure state, rather than trait, body dissatisfaction [43]. Responses were measured using a slider scale rather than a Likert scale to minimize the likelihood that participants would remember and reproduce their pre-training responses when completing the post-training scale. The position of the slider represented unseen response options ranging from 0 to 100 (0 being ‘Very satisfied’ and 100 being ‘Very dissatisfied’). Body dissatisfaction scores were calculated by summating the responses for all 18 items; therefore, possible body dissatisfaction scores ranged between 0 and 1800 with higher scores indicating greater body dissatisfaction. All participants completed the body shape satisfaction scale pre- and post-training. Cronbach alpha values for this version of the experiment were 0.94 at pre-training and 0.96 at post-training, indicating excellent internal consistency for the scale. Change in body dissatisfaction (ΔBD) was calculated by subtracting pre-training body dissatisfaction scores from post-training body dissatisfaction scores. A positive (negative) ΔBD meant that participants' body dissatisfaction increased (decreased) after training.

### 2.4. Procedure

Participants signed up to the experiment remotely using their chosen recruitment platform (Prolific or Macquarie University's study sign-up system), which directed participants to the experiment via a hyperlink. The experiment was hosted on the Gorilla Experiment Builder (www.gorilla.sc [44]). We specifically used the Gorilla Experiment Builder to host the experiment because although the platform has a reaction time recording latency of around 80 ms, this latency is relatively consistent for all operating systems and device types [45]. The platform also has very good temporal precision for recording reaction times (approx. equal to 8.25 ms) and is often more precise than other online experiment platforms [45]. The Gorilla Experiment Builder has previously replicated the findings of similar reaction time studies using a variety of online settings, equipment and Internet connection types [44]. Participants could only access the experiment if they used a laptop or desktop computer, and not a smartphone or tablet, to ensure they were able to make keyboard responses. The experiment took approximately 45 min to complete, and all experimental instructions were presented on the computer screen. The experiment expired after 90 min to minimize the likelihood of participants taking breaks during the experiment.

Participants were first asked to confirm whether they had previously completed the experiment via an alternative platform (Prolific or Macquarie University's study sign-up system), or whether they had previously completed other experiments presented in this paper. Participants were then asked to provide demographic information, including their height and weight so we could calculate self-reported body mass index (BMI; kg m^−2^). Participants then completed the pre-training body dissatisfaction questionnaire followed by three practice PSN trials and the 10 pre-training PSN trials. Body stimuli for the practice PSN trials were three target identities selected at random for each participant from the pre- and post-training PSN target identities. Participants then completed 10 practice dot probe trials (which were identical to the pre- and post-training dot probe trials), followed by the 80 pre-training dot probe trials, followed by the 360 training dot probe trials. Participants then completed the post-training body dissatisfaction questionnaire, followed by the 80 post-training dot probe trials and the 10 post-training PSN trials interwoven in the same block, i.e. one PSN trial, then eight dot probe trials, then one PSN trial and so on. The interwoven order was counterbalanced so that half of participants started with one PSN trial (followed by eight dot probe trials, and so on) and half of participants started with eight dot probe trials (followed by one PSN trial, and so on). We used this interwoven order because the post-training dot probe trials directed participants' attention towards both high- and low-fat body stimuli, which could potentially reduce adaptation induced by the training dot probe trials. We aimed for the interwoven order to minimize order effects and increase the likelihood of detecting an effect for body size adaptation.

### 2.5. Data analysis

Data were initially screened at a participant level. No participants reported previously completing the experiment via an alternative platform or completing one of the other experiments presented in this paper. One participant had missing data and six participants responded correctly on fewer than 60% of either the pre- or post-training dot probe trials, so we excluded these participants and recruited seven replacement participants.

The following analyses were conducted on R v. 4.0.5 [46]. First, to check whether our results replicated previous cross-sectional dot probe studies reporting a positive relationship between body dissatisfaction and attentional bias towards low-fat bodies, we conducted correlation analysis on the pre-training attentional bias scores and pre-training body dissatisfaction scores collapsed across conditions. Next, to test our hypotheses, we conducted six confirmatory frequentist one-sample *t*-tests to compare participants’ ΔAB, ΔPSN and ΔBD against a value of 0 separately for each condition (high-fat and low-fat). We specifically chose not to compare attentional bias scores between participants, because doing so could introduce reaction time noise from participants using different devices and Internet connection types. Due to the non-normal distribution of many variables in this study (see electronic supplementary material), we used bootstrapping of the mean to estimate *p*-values and 95% confidence intervals [47]. Bootstrapped statistics were bias-corrected accelerated and computed using the R package wBoot with 2000 iterations [48]. We used the Holm–Bonferroni method to assess the results of the six tests [49]; therefore, our lowest alpha criterion was 0.008 (0.05/6).

To further test our hypotheses, we conducted six exploratory Bayesian one-sample *t*-tests using the R package BayesFactor to determine the likelihood of the alternative hypotheses in relation to their corresponding null hypotheses for each condition (Cauchy prior, *r* = 0.707 [50]). Unlike frequentist one-sample *t*-tests, Bayesian one-sample *t*-tests can be used to determine whether there is evidence for the null hypothesis or whether the data are too insensitive to interpret [51]. For each test, the alternative hypothesis assumed that the true mean of the sample was not equal to zero, while the null hypothesis assumed that the true mean of the sample was equal to zero. A Bayes factor between 3 and 10 was interpreted as moderate evidence for the alternative hypothesis, a Bayes factor between 1 and 3 was interpreted as anecdotal evidence for the alternative hypothesis, a Bayes factor between 1/3 and 1 was interpreted as anecdotal evidence for the null hypothesis, and a Bayes factor between 1/3 and 1/10 was interpreted as moderate evidence for the null hypothesis [52,53]. Lastly, we conducted exploratory sensitivity analyses and ran the one-sample *t*-tests without bootstrapping of the mean and with outliers removed from the data. Following the approach used by Dondzilo *et al*. [22], outliers were defined as values more than three standard deviations above or below the mean.

### 2.6. Results

The correlation analyses on the pre-training data provided no clear evidence to suggest that attentional bias scores correlated with body dissatisfaction scores (*r*_148_ = 0.05, *p* = 0.575). The results of the frequentist and Bayesian one-sample *t*-tests are presented in table 1. The frequentist one-sample *t*-tests provide no clear evidence to suggest that participants' ΔAB, ΔPSN or ΔBD differed from 0 for either condition. All Bayes factors demonstrated moderate evidence for the null hypothesis, except for ΔPSN in the low-fat condition which only provided anecdotal evidence for the null hypothesis. These results remained consistent when we reran the one-sample *t*-tests without bootstrapping of the mean and when we removed outliers from the data (see electronic supplementary material).

**Table 1.** Experiment 1 results for the one-sample *t*-tests and Bayes factors (BF_10_) comparing change in attentional bias (ΔAB), change in point of subjective normality (ΔPSN) and change in body dissatisfaction (ΔBD) against a value of 0 for each attention training condition (Cauchy prior, *r* = 0.707). Bootstrap resampling was used to estimate *p*-values and 95% confidence intervals. *N* = 150 (75 participants per condition). CI = confidence interval.

condition	ΔAB						ΔPSN						ΔBD					
	*M* [95% CI]	s.d.	*t*	*p*	*d*	BF_10_	*M* [95% CI]	s.d.	*t*	*p*	*d*	BF_10_	*M* [95% CI]	s.d.	*t*	*p*	*d*	BF_10_
high-fat	1.46 [−12.24, 14.28]	58.35	0.22	0.849	0.03	0.13	−0.20 [−0.78, 0.37]	2.54	−0.68	0.504	0.08	0.16	−35.84 [−128.50, 1.35]	247.13	−1.26	0.066	0.15	0.27
low-fat	8.28 [−3.53, 21.22]	58.00	1.24	0.166	0.14	0.26	−0.41 [−0.95, 0.10]	2.37	−1.50	0.110	0.17	0.37	−9.85 [−39.53, 8.87]	103.49	−0.82	0.299	0.10	0.18

### 2.7. Discussion

The results for Experiment 1 showed that participants trained to attend to low (high)-fat body stimuli did not exhibit a greater attentional bias to low (high)-fat body stimuli, perceive lower (higher) fat body stimuli as ‘normal’ or exhibit higher (lower) body dissatisfaction as a result of the attention training. These results do not support Hypotheses 1–3 and indicate that the training dot probe task did not effectively modify participants' attention towards high- or low-fat body stimuli. Because the training dot probe task failed to modify attention, we cannot determine whether attention to low- or high-fat bodies is likely to have a causal effect on body size adaptation or body dissatisfaction. One possible explanation for the failure of the training dot probe task to modify attention is that the experiment was completed by participants online and therefore we had little control over the experiment setting. Factors such as noise, distractions, screen size and the absence of an experimenter may have prevented some participants from fully engaging in the experiment. A commonly discussed advantage of attentional bias modification tasks is they can be completed by patients online in a home setting; however, some research suggests that the tasks may be more effective at manipulating attention in a laboratory setting [32].

## 3. Experiment 2

To test whether the effects of the training dot probe task were influenced by the experiment setting, we repeated Experiment 1 in a laboratory setting and compared the results to Experiment 1. In addition to our original three hypotheses, we hypothesized that (4) participants trained in a laboratory setting would show greater changes in attentional bias, body size adaptation and body dissatisfaction than participants trained online. The experiment methodology was almost identical to Experiment 1; however, we introduced minor methodological changes to adapt the experiment to a laboratory setting. The experiment was preregistered with Experiment 1 on the Open Science Framework (doi:10.17605/OSF.IO/TJPZB).

### 3.1. Participants

An *a priori* power analysis (G*Power v. 3.1.9.2 [38]) showed we had 80% power for our main analyses (one-sample *t*-tests) to detect a medium effect size for our primary outcome (ΔAB) at an alpha level of 5% with a sample size of 70 participants. Participants were 70 White/European origin women aged 18–35 years (35 participants per condition; *M*_age_ = 21.07, s.d. = 3.50; *M*_BMI_ = 23.63, s.d. = 5.13). We placed no restrictions on the participant's country of residence. Participants were recruited using advertisements on Macquarie University's study sign-up system, flyers posted around the local area, social media posts to local psychology groups, and through friends of the researchers. Participants could choose to be reimbursed with either course credit or $20 (AUD).

### 3.2. Stimuli

The experiment was presented on a 35.3 × 26.5 cm display with a resolution of 1292 × 969 pixels. Participants viewed the experiment at an approximate distance of 60 cm; therefore, the stimuli sizes were approximately the same for all participants (dot probe tasks: 10.58 × 15.89 cm, 387 × 581 pixels, 10.08 × 15.09° degrees of visual angle; method of adjustment tasks: 12.33 × 18.51 cm, 451 × 677 pixels, 11.73 × 17.54°).

### 3.3. Measures

#### 3.3.1. Body dissatisfaction

We used the same modified version of the body shape satisfaction scale as Experiment 1 [42]. Cronbach alpha values were 0.95 at both pre-training and post-training, indicating excellent internal consistency for the scale.

### 3.4. Procedure

The procedure was almost identical to Experiment 1; however, participants completed the experiment using Google Chrome on a desktop computer (ASUS ET2322; 60 Hz) with a USB port keyboard (125 Hz) in the presence of an experimenter in the Department of Psychology, Macquarie University. Height and weight were measured with a tape measure and a Tanita SC-330 body composition analyser to calculate each participant's BMI.

### 3.5. Data analysis

Data screening and analysis were identical to Experiment 1, except in the following respects. One participant reported having previously completed Experiment 1; therefore, we excluded this participant and recruited a replacement participant. No participants needed to be excluded for having missing data or responding correctly on less than 60% of either the pre- or post-training dot probe trials. To test Hypothesis 4, we tested whether effect sizes for each variable (ΔAB, ΔPSN and ΔBD) separated by condition were larger for the laboratory setting (Experiment 2) than the online setting (Experiment 1). We conducted bootstrap resampling using the R package bootES with 2000 samples to estimate 95% confidence intervals for each effect size (Cohen's *d*) [54]. We inferred there being evidence for Hypothesis 4 if the effect sizes in Experiment 2 were larger than their corresponding effect sizes in Experiment 1 with non-overlapping 95% confidence intervals.

### 3.6. Results

The correlation analyses on the pre-training data provided no clear evidence to suggest that attentional bias scores correlated with body dissatisfaction scores (*r*_68_ = −0.09, *p* = 0.440). The results of the frequentist and Bayesian one-sample *t*-tests are presented in table 2. For participants in the high-fat condition, the results of the frequentist one-sample *t*-tests provide strong evidence for participants increasing their attention to high-fat bodies as a result of the attention training, and the Bayes factor provides moderate support for this hypothesis. However, the frequentist one-sample *t*-tests provided no clear evidence to suggest these participants' ΔPSN or ΔBD differed from 0. The Bayes factors’ support for the null hypothesis was anecdotal for ΔPSN and moderate for ΔBD. For participants in the low-fat condition, the frequentist one-sample *t*-tests provide no clear evidence to suggest participants' ΔAB, ΔPSN or ΔBD differed from 0. The Bayes factors’ support for the null hypothesis was anecdotal for ΔPSN and moderate for ΔAB and ΔBD. These results remained consistent when we reran the one-sample *t*-tests without bootstrapping of the mean and when we removed outliers from the data (see electronic supplementary material).

**Table 2.** Experiment 2 results for the one-sample *t*-tests and Bayes factors (BF_10_) comparing change in attentional bias (ΔAB), change in point of subjective normality (ΔPSN) and change in body dissatisfaction (ΔBD) against a value of 0 for each attention training condition (Cauchy prior, *r* = 0.707). Bootstrap resampling was used to estimate *p*-values and 95% confidence intervals. *N* = 70 (35 participants per condition). CI = confidence interval.

condition	ΔAB						ΔPSN						ΔBD					
	*M* [95% CI]	s.d.	*t*	*p*	*d*	BF_10_	*M* [95% CI]	s.d.	*t*	*p*	*d*	BF_10_	*M* [95% CI]	s.d.	*t*	*p*	*d*	BF_10_
high-fat	−22.76 [−39.77, −8.21]	47.71	−2.82	<0.001	0.48	5.22	−0.51 [−1.34, 0.28]	2.49	−1.22	0.209	0.21	0.36	0.54 [−20.32, 23.54]	69.06	0.05	0.997	0.01	0.18
low-fat	6.31 [−6.05, 21.10]	40.75	0.92	0.301	0.16	0.27	−0.89 [−2.02, −0.12]	2.71	−1.94	0.018	0.33	0.97	2.23 [−17.46, 23.12]	64.06	0.21	0.854	0.04	0.18

**Figure 3.**
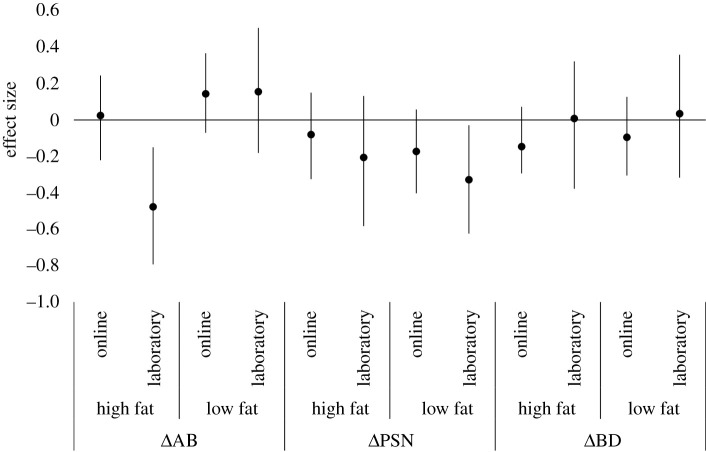
Effect sizes (Cohen's *d*) for change in attentional bias (ΔAB), change in point of subjective normality (ΔPSN) and change in body dissatisfaction (ΔBD) separated by attention training condition for the online setting (Experiment 1) and the laboratory setting (Experiment 2). Bootstrap resampling was used to estimate 95% confidence intervals.

The effect sizes and their bootstrapped 95% confidence intervals for each variable and condition are presented in figure 3 with their corresponding effect sizes from Experiment 1. When looking at each variable and condition, the 95% confidence intervals for the online setting (Experiment 1) and laboratory setting (Experiment 2) overlapped, demonstrating no clear evidence that the experiment setting influenced the size of effects of the training dot probe task on ΔAB, ΔPSN or ΔBD. A near exception was ΔAB in the high-fat condition where the 95% confidence interval overlap between the online and laboratory setting was only marginal. The ΔAB effect size for the high-fat condition in the laboratory setting was medium in size [55] and the 95% confidence intervals did not overlap with zero, supporting the suggestion that this training dot probe task effectively increased attention towards high-fat bodies. By contrast, the ΔAB effect size for the high-fat condition in the online setting was very small in size and had 95% confidence intervals overlapping zero. These results could point to a possible effect of experiment setting, with larger ΔAB effects for the high-fat condition in the laboratory setting than the online setting; however, given that there was still an overlap between the 95% confidence intervals for the laboratory and online effect sizes, there is little evidence for this effect.^1^ These results remained consistent when we removed outliers from the data (see electronic supplementary material).

### 3.7. Discussion

The results for Experiment 2 showed that participants trained to attend towards low-fat body stimuli did not exhibit a greater attentional bias to low-fat body stimuli, perceive lower fat body stimuli as ‘normal’ or exhibit higher body dissatisfaction as a result of the attention training. These results do not support Hypotheses 1–3 and indicate that the training dot probe task did not effectively modify participants' attention to low-fat body stimuli. By contrast, participants trained to attend to high-fat bodies did increase their attention to high-fat bodies, in support of Hypothesis 1. However, participants in this condition did not perceive higher fat body stimuli as ‘normal’ or exhibit lower body dissatisfaction as a result of the training, and therefore these results do not support Hypotheses 2 and 3. The training dot probe task appeared to increase participants' attention to high-fat body stimuli without influencing their perceptions of a ‘normal’ body size or body dissatisfaction.

The results for this experiment indicate that the training dot probe task was effective at modifying attention towards high-fat bodies in a laboratory setting, unlike the online training dot probe task conducted in Experiment 1. However, the overlapping 95% confidence intervals around the effect sizes did not provide convincing evidence for an effect of experiment setting and therefore did not support Hypothesis 4. As a result, we are cautious to dismiss the null findings of Experiment 1 as being a consequence of the online setting. Another potential factor contributing to the null findings of Experiment 1 was the stimulus onset -asynchrony (SOA) during the dot probe task (i.e. the time period between the onset of the presentation of body stimuli and the onset of the probe presentation). For Experiments 1 and 2, we used a 500 ms SOA to be consistent with other dot probe studies that have successfully modified participants' attention towards low-fat bodies [36,58]. However, the dot probe task may be more reliable using a shorter SOA (100 ms), which limits the number of overt and covert attentional shifts participants can make during the presentation of stimuli. Shorter SOAs are thought to increase the reliability of the dot probe task as a measure of attentional bias, because participants who have their attention captured initially by the target stimulus do not have time to redistribute their attention away from the target stimulus before the probe onset [59].

## 4. Experiment 3

To test whether the effects of the training dot probe task are influenced by SOA length, we repeated Experiment 1 using a shorter SOA. Due to restrictions on face-to-face data collection in response to the Coronavirus pandemic, we chose to conduct Experiment 3 in an online setting and compare the results with Experiment 1. The experiment was identical to Experiment 1 except that the SOA during the pre-training, training and post-training dot probe tasks was reduced from 500 ms to 100 ms. Therefore, each dot probe trial started with a 1000 ms fixation, followed by one high- and one low-fat body stimulus presented simultaneously for 100 ms, followed by the probe (p or q) which participants had to identify as quickly and accurately as possible.

By shortening the SOA of the dot probe task, we aimed to increase the reliability of the task as a measure of attentional bias by limiting the number of overt and covert attentional shifts during the stimuli presentation [59]. However, a 100 ms SOA during the training dot probe trials may also influence the likelihood of participants adapting to their target stimulus. Timescales for body size after-effects are currently unknown; however, after-effects generally decay faster after shorter adaptation periods [60]. Therefore, a 100 ms SOA may reduce the likelihood of participants displaying measurable body size after-effects. On the other hand, a training dot probe task with a 500 ms SOA might only train participants to shift their attention towards the target stimulus during the later stages of the stimulus presentation, meaning that participants could still attend to the opposing stimulus in the earlier stages of the stimulus presentation. If this is the case, then a 100 ms SOA might actually increase the likelihood of body size after-effects, because participants only have time to attend towards one stimulus prior to probe onset and will spend more time attending towards the target stimulus relative to the opposing stimulus. Therefore, in addition to our original three hypotheses, we hypothesized that (5) participants completing the experiment with a 100 ms SOA would show greater changes in attentional bias, body size adaptation and body dissatisfaction than participants completing the experiment with a 500 ms SOA. This experiment was preregistered on the Open Science Framework (doi:10.17605/OSF.IO/5NS2G).

### 4.1. Participants

We recruited 150 White/European origin women aged 18–35 years (75 participants per condition; *M*_age_ = 20.51, s.d. = 3.53; *M*_BMI_ = 23.63, s.d. = 5.75). We placed no restrictions on the participant's country of residence. All participants were recruited through the Macquarie University's study sign-up system and reimbursed with course credit.

### 4.2. Measures

#### 4.2.1. Body dissatisfaction

We used the same modified version of the body shape satisfaction scale as the previous experiments [42]. Cronbach alpha values were 0.94 at pre-training and 0.96 at post-training, indicating excellent internal consistency for the scale.

### 4.3. Data analysis

Data screening and analysis procedures were identical to Experiment 1. One participant reported having previously completed Experiment 2, one participant had missing data and two participants responded correctly on less than 60% of either the pre- or post-training dot probe trials, so we excluded these participants and recruited four replacement participants. To test Hypothesis 5, we analysed the effect of SOA by comparing ΔAB, ΔPSN and ΔBD for Experiment 1 (SOA = 500 ms) and Experiment 3 (SOA = 100 ms). We conducted three frequentist 2 × 2 between-participants ANOVAs—one ANOVA for each dependent variable (ΔAB, ΔPSN and ΔBD). For each ANOVA, the first independent variable was the body size targeted in the attention training (high versus low fat). The second independent variable was the SOA of the body stimuli during the dot probe tasks (500 ms versus 100 ms). We inferred there being evidence to support Hypothesis 5 if the interaction for each ANOVA had a *p* < 0.05 and participants trained with a 100 ms SOA to attend towards low (high)-fat bodies demonstrated a higher (lower) ΔAB, a lower (higher) ΔPSN and a higher (lower) ΔBD than participants trained with a 500 ms SOA. We also conducted three Bayesian versions of each ANOVA. Bayes factors were computed using the R package BayesFactor [50] to compare the interaction models against the null intercept-only models. We used the same criteria as described previously to evaluate whether each Bayes Factor provided support for the null intercept-only model or the interaction models [52,53].

### 4.4. Results

The correlation analyses on the pre-training data provided no clear evidence to suggest that attentional bias scores correlated with body dissatisfaction scores (*r*_148_ = −0.01, *p* = 0.886). The results of the frequentist and Bayesian one-sample *t*-tests are presented in table 3. The frequentist one-sample *t*-tests provide no clear evidence to suggest that participants' ΔAB, ΔPSN or ΔBD differed from 0 for either condition. All Bayes factors demonstrated moderate evidence for the null hypothesis. These results remained consistent when we reran the one-sample *t*-tests without bootstrapping of the mean and when we removed outliers from the data (see electronic supplementary material).

**Table 3.** Experiment 3 results for the one-sample *t*-tests and Bayes factors (BF_10_) comparing change in attentional bias (ΔAB), change in point of subjective normality (ΔPSN) and change in body dissatisfaction (ΔBD) against a value of 0 for each attention training condition (Cauchy prior, *r* = 0.707). Bootstrap resampling was used to estimate *p*-values and 95% confidence intervals. *N* = 150 (75 participants per condition). CI = Confidence interval.

condition	ΔAB						ΔPSN						ΔBD					
	*M* [95% CI]	s.d.	*t*	*p*	*d*	BF_10_	*M* [95% CI]	s.d.	*t*	*p*	*d*	BF_10_	*M* [95% CI]	s.d.	*t*	*p*	*d*	BF_10_
high-fat	−9.24 [−23.42, 8.68]	71.78	−1.12	0.306	0.13	0.23	−0.23 [−0.73, 0.25]	2.20	−0.91	0.353	0.11	0.19	3.52 [−13.18, 20.83]	80.22	0.38	0.735	0.04	0.14
low-fat	−18.06 [−57.05, 1.79]	115.28	−1.36	0.073	0.16	0.31	−0.12 [−0.62, 0.44]	2.33	−0.46	0.724	0.05	0.14	11.51 [−5.93, 30.44]	79.63	1.25	0.212	0.15	0.27

The results of the frequentist 2 × 2 ANOVAs for ΔAB, ΔPSN and ΔBD did not provide evidence for an interaction effect between SOA and condition (table 4). Therefore, the results do not support Hypothesis 5. There was some evidence for a main effect of SOA on ΔAB with participants demonstrating a more negative ΔAB with a 100 ms SOA than a 500 ms SOA. These results indicate that participants may have been more likely to increase their attention towards high-fat bodies as a result of the training with a 100 ms SOA when compared with a 500 ms SOA, regardless of the body size participants were trained to attend toward. However, the partial eta squared for the SOA main effect was small and the *p*-value increased substantially when outliers were removed (to *p* = 0.225; see electronic supplementary material), indicating that this result may have been driven by a small number of participants.

**Table 4.** The results of the three frequentist 2 × 2 between-participants ANOVAs testing the effects of SOA (100 ms versus 500 ms) and attention training condition (high fat versus low fat) in the online experiments on change in attentional bias (ΔAB), change in point of subjective normality (ΔΔPSN) and change in body dissatisfaction (ΔBD). *N* = 300.

	d.f.	ΔAB			ΔPSN			ΔBD		
		*F*	*p*	$\eta_{p}{\;}^{2}$	*F*	*p*	$\eta_{p}{\;}^{2}$	*F*	*p*	$\eta_{p}{\;}^{2}$
predictor										
SOA	1	4.08	0.044	0.01	0.22	0.639	0.00	3.27	0.072	0.01
condition	1	0.01	0.913	0.00	0.03	0.853	0.00	1.02	0.313	0.00
SOA × condition	1	0.73	0.394	0.00	0.34	0.558	0.00	0.29	0.592	0.00

The results of the three Bayesian 2 × 2 between-participants ANOVAs demonstrate strong support for the null intercept-only model when compared with the interaction model for ΔAB, ΔPSN and ΔBD (table 5). When compared with the remaining main effect models, support for the null intercept-only model ranged from strong to anecdotal. Overall, the results of the frequentist and Bayesian ANOVAs indicate that SOA had no effect on ΔAB, ΔPSN or ΔBD.

**Table 5.** Bayes factors (BF_10_) for the three Bayesian 2 × 2 between-participants ANOVAs testing the effects of SOA (100 ms versus 500 ms) and attention training condition (high fat versus low fat) in the online experiments on change in attentional bias (ΔAB), change in point of subjective normality (ΔPSN) and change in body dissatisfaction (ΔBD). Models are compared against the null intercept-only model. *N* = 300.

model	ΔAB	ΔPSN	ΔBD
SOA	0.89	0.14	0.60
condition	0.13	0.13	0.21
SOA + condition	0.11	0.02	0.13
SOA + condition + SOA × condition	0.03	0.00	0.02

### 4.5. Discussion

The results for Experiment 3 showed that participants trained to attend towards low (high)-fat body stimuli did not exhibit a greater attentional bias to low (high)-fat body stimuli, perceive lower (higher) fat body stimuli as ‘normal’ or exhibit higher (lower) body dissatisfaction as a result of the attention training. These results do not support Hypotheses 1–3 and indicate that the training dot probe task did not effectively modify participants' attention towards high- or low-fat body stimuli. Because the training dot probe task did not modify attention, we cannot determine whether attention to low- or high-fat bodies is likely to have a causal effect on body size adaptation or body dissatisfaction. We aimed to increase the reliability of this dot probe task by using a shorter SOA (100 ms) to limit the number of overt and covert attentional shifts participants could make during the stimuli presentation [59]. However, when we compared the results of Experiment 3 to Experiment 1, the results did not support Hypothesis 5. Participants trained with a 100 ms SOA to attend towards low (high)-fat bodies did not demonstrate a higher (lower) ΔAB, a lower (higher) ΔPSN or a higher (lower) ΔBD than participants trained with a 500 ms SOA. Therefore, shortening the SOA from 500 ms to 100 ms did not influence the effects of the training dot probe task.

## 5. General discussion

We conducted three experiments to investigate whether a dot probe attention training task influenced participants’ attention towards high- versus low-fat bodies, body size adaptation and body dissatisfaction. We found evidence to suggest that the dot probe task was effective at modifying attention towards high-fat bodies for participants in a laboratory setting (Experiment 2). However, participants in this condition did not perceive bodies as smaller as a result of the attention training, i.e. they did not adapt to the high-fat body stimuli. Neither did participants exhibit lower body dissatisfaction as a result of the training. Therefore, the training dot probe task appeared to increase participants' attention towards high-fat body stimuli without inducing body size after-effects or influencing body dissatisfaction.

The lack of change in body dissatisfaction for this condition is perhaps unsurprising, because changes in body dissatisfaction might be contingent on body size adaptation. This suggestion is supported by studies showing the co-occurrence of body size after-effects and changes in body dissatisfaction. For example, Bould and colleagues found that women exposed to unfamiliar high-fat body stimuli reduced their size estimations for subsequently presented body stimuli, indicating that they adapted to the high-fat body stimuli. The participants also reported reduced body dissatisfaction, which may have been a consequence of the body size adaptation [27]. On the other hand, Stephen *et al*. [28] directed participants’ attention towards high-fat bodies and participants adapted to the high-fat bodies without reporting reduced body dissatisfaction. Therefore, body size after-effects might be necessary but not sufficient to induce changes in body dissatisfaction.

The lack of body size after-effects for this condition is more surprising, because Stephen *et al*. [28] found body size after-effects depended on the body size the participants were instructed to attend towards. We used the same body stimuli as Stephen and colleagues and therefore expected to see similar body size after-effects. One possible explanation for this discrepancy is that fixations are required to sufficiently induce measurable body size after-effects. Stephen and colleagues used eye-tracking to confirm they modified participants' overt attention and found that participants fixated more on the body size they were instructed to attend towards. By contrast, the dot probe task can be completed without eye-movements and therefore is thought to measure covert attention [37]. The dot probe task for Experiment 2 used a 500 ms SOA, which is sufficient for participants to make saccades and, as these were not measured, we cannot completely rule eye-movements out. However, our comparison of Experiment 1 and 3 indicated there was no effect of SOA (500 ms versus 100 ms) on ΔAB and, given that we know eye-movements are not possible using a 100 ms SOA [61], it seems unlikely that they were driving the increase in attention to high-fat bodies in Experiment 2. Therefore, participants’ fixation durations over the course of the training may have been insufficient to induce measurable body size after-effects.

If fixations are required to induce body size after-effects, this would imply that body size after-effects, like motion after-effects, are retinotopic, i.e. they only occur when the adaptation and test stimuli appear on the same place on the retina [62,63]. In our experiments, the adaptation stimuli were presented during the training dot probe task on the left and right side of the screen, whereas the test stimuli were presented during the pre- and post-training method of adjustment tasks in the centre of the screen. Therefore, if participants did not make fixations towards the body stimuli during the training dot probe task, then the adaptation and test stimuli would have probably appeared in different places on the retina, which may have prevented adaptation. However, evidence suggests that body size after-effects are not retinotopic and instead, like face after-effects [64], they use an object-centred frame of reference [30]. Brooks *et al*. [30] found that people displayed body size after-effects even when the orientation of the adaptation and test stimuli differed, indicating that body size after-effects are unlikely to be localized to a specific point on the retina and are instead likely to be processed by cells with larger receptive fields. Therefore, body size after-effects are possible even when adaptation and test stimuli appear at different points on the retina, meaning body size after-effects should have been possible without participants fixating on the adaptation stimuli.

Another possible explanation for this discrepancy is the difference in timescales for the adaptation periods. The training dot probe task for Experiment 2 presented the body stimuli for 500 ms per trial and participants completed 360 training trials; therefore, the adaptation stimuli were presented for a total time of three minutes. However, this adaptation period was not continuous and instead was interrupted by periods where the body stimuli were not presented on the screen, e.g. during the fixation and response periods, and the five 15 s breaks. Therefore, the entire duration of the training dot probe task was longer than 3 min, and most participants took approximately 15 min to complete the task. By contrast, Stephen *et al*. [28] presented body stimuli to participants continuously for a 2 min adaptation period, and during the post-adaptation test phase, participants were presented with ‘top-up’ adaptation stimuli to maintain their adaptation. Timescales for body size after-effects are currently unknown; however, after-effects generally decay over time unless a person is re-exposed to the adaptation stimulus [60]. Therefore, unlike the body size after-effects induced by Stephen *et al*. [28], any body size after-effects induced by our training dot probe task could have decayed by the time participants completed the post-training measures.

The remaining results from our three experiments showed that participants trained to attend to low (high)-fat body stimuli did not exhibit a greater attentional bias to low (high)-fat body stimuli, perceive lower (higher) fat body stimuli as ‘normal’ or exhibit higher (lower) body dissatisfaction as a result of the attention training. These results indicate that the training dot probe task did not effectively modify participants' attention towards high- or low-fat body stimuli for either condition. Given the training dot probe task failed to modify participants’ attention for these conditions, the absence of body size after-effects and changes in body dissatisfaction is in line with expectations, because these variables were only hypothesized to change as a result of a change in attention towards high- and low-fat body stimuli.

The absence of a change in attention contrasts with previous dot probe attention training studies. For example, Dondzilo and colleagues used a dot probe task to effectively train participants to attend towards or avoid low-fat bodies [36,58]. The discrepancy in results is consistent with the finding that effect sizes are smaller for preregistered studies than non-preregistered studies [65]. Although we adjusted for the inflation of effect sizes in our *a priori* power analyses, this adjustment may not have been sufficient for our experiments to detect small effect sizes, especially if the effects were too small to be detected using the temporal precision of our experiment platform (approx. equal to 8.25 ms; [45]). Alternatively, another possible reason for the discrepancy is that Dondzilo and colleagues presented participants with the low-fat body stimulus alongside an abstract art image, as opposed to a high-fat body stimulus as used in the present experiments. Therefore, Dondzilo and colleagues may have modified participants' attention towards bodies generally, rather than specifically to low-fat bodies. The high- and low-fat body stimuli used in the present experiments differed in apparent fat mass by 24 kg; however, the stimuli may not have been visually contrasting enough to induce a measurable attention training effect. More extreme body stimuli may have been more likely to capture the participants’ attention and may also have been a more realistic representation of the range of body sizes in the general population.

When evaluating our body stimuli, we should also consider the results of the correlation analyses on the pre-training data, which were also discrepant with previous cross-sectional dot probe studies [22–24]. In contrast with the aforementioned studies, we did not find evidence to support the positive association between body dissatisfaction and attentional bias towards low-fat bodies. Two of these studies used similar stimulus pairs to the present experiments i.e. one small and one large body size; however, the BMI of these stimulus pairs were more extreme than the stimuli used in the present experiments [23,24]. Therefore, it is possible that the restricted BMI range of our body stimuli prevented us from sufficiently modifying attentional bias. However, our results are more in line with a study by Glauert *et al*. [66] who conducted a similar dot probe task using body stimuli with a more extreme BMI range, estimated as 11.7 and 30.4 units. They found no evidence for a relationship between body dissatisfaction and attentional bias towards low-fat bodies. In a subsequent systematic review, Rodgers and DuBois suggested that Glauert and colleagues did not find a relationship because the body stimuli were unrelatable [67]. Glauert and colleagues used unclothed body stimuli that appeared emaciated and far thinner than we would expect to see in mainstream media, and therefore they were considered less likely to attract attention from people with high body dissatisfaction. Therefore, it is possible that future dot probe research may be more effective at modifying attention using body stimuli representing a BMI range that is less restricted than the body stimuli used in the present experiments, but not quite as extreme as the body stimuli used by Glauert and colleagues.

Another potential explanation for these contrasting results is the poor reliability of the dot probe task as a measure of attentional bias. The dot probe task has previously been shown to have poor internal consistency and test–retest reliability [59,68–70], which may explain why studies using the dot probe task report inconsistent results for the relationship between body dissatisfaction and attentional bias towards low-fat bodies. By contrast, studies using eye-tracking measures consistently report a positive relationship [18–21]. Given the poor reliability of the dot probe task as a measure of attentional bias, we should interpret our results for ΔAB with caution. It is possible, for example, that the results indicating that participants increased their attention towards high-fat bodies in the laboratory setting (Experiment 2) were a Type 1 error. If the attention training did not actually modify attention in this condition, this would provide an additional explanation for the lack of body size after-effects and change in body dissatisfaction.

On the other hand, it is also possible that the five remaining null results for ΔAB were Type 2 errors. Therefore, the attention training may have worked; however, the dot probe task was not reliable enough to detect changes in attentional bias. This suggestion is supported by recent research using event-related potentials (ERPs), which are a more reliable measure of attentional bias than the dot probe task [71] and are more consistently modulated by attention training dot probe tasks [72]. However, we think this interpretation is less likely, given that our experiments produced five null results out of six for ΔAB and Bayesian analyses demonstrated moderate support for each of the five null hypotheses. The dot probe task used here was clearly ineffective at producing a reliable change in attention towards high- versus low-fat bodies, and this is probably the reason for the lack of body size after-effects and change in body dissatisfaction.

## 6. Conclusion

In conclusion, training participants to attend towards high- versus low-fat bodies using a dot probe task was ineffective at inducing body size after-effects and changes in body dissatisfaction. Given the training dot probe task seemed largely ineffective at modifying attention, it is unsurprising that the task did not elicit the predicted body size after-effects or changes in body dissatisfaction. The only exception was for participants trained to attend towards high-fat bodies in the laboratory setting (Experiment 2). These participants increased their attention towards high-fat bodies, as measured on the dot probe task; however, this change in attentional bias did not lead participants to perceive higher fat body stimuli as more ‘normal’ or report reduced body dissatisfaction. These findings could be explained by the need for fixations to elicit body size after-effects, the short duration of any elicited body size after-effects, the restricted BMI range of our body stimuli or the poor reliability of dot probe task as a measure of attentional bias. Together, our findings suggest the training dot probe task used in the present research is unlikely to be an effective method for modifying body image disturbances in young adult women of White/European origin. Future research using training dot probe tasks to modify attention should avoid additionally using the dot probe task to measure change in attentional bias. Instead, researchers should use more reliable measures of attentional bias (e.g. ERPs) to assess the effectiveness of the attention modification.

## Footnotes

1 We are aware that our preregistered inference criteria of non-overlapping 95% confidence intervals for Hypothesis 4 may be quite conservative. For ΔAB in the high-fat condition, the 95% confidence interval overlap for the online and laboratory experiment was less than half the average margin of error (proportion overlap = 0.25). This implies the *p*-value for the difference between effect sizes would be between 0.01 and 0.05 [56], which could be interpreted as evidence for an effect of experiment setting. However, this evidence would be weak at best [57] and unlikely to affect our overall conclusions.

## Data accessibility

Our metadata, analysis code, and study documents are publicly available via the University of Bristol's data.bris research data repository: https://doi.org/10.5523/bris.eb3tz0onvuld2qwqrxc1z3q7d [73]. The main part of this material includes our analysis code and data dictionary, which would allow interested researchers to understand exactly how we used R to calculate each statistic reported in our manuscript. A full replication of the analysis code with our data sheet will not be possible, because we cannot also share the data sheet which we imported into R. Instead, our aim here is to ensure readers can fully understand each analysis step even without being able to conduct a full replication. Unfortunately, the data generated during the study cannot be made accessible because our consent forms explicitly said that we would keep the data solely within our research team; however, we provide a clearly labelled synthetic dataset alongside our analysis code to allow interested researchers to see the code in action as a proof of principle. The editorial office has confirmed that the journal will make an exception to data sharing requirements on the basis that providing the dataset would be in violation of consent forms and present unacceptable ethical concerns.
